# Effects of Percutaneous Electrical Stimulation on Chronic Neuropathic Pain: A Retrospective Observational Study

**DOI:** 10.7759/cureus.79203

**Published:** 2025-02-18

**Authors:** Fabio Ferraiuolo, Paola Camelli, Giuliano Chiappini, Laura Tommasi, Maurizio Massetti

**Affiliations:** 1 Emergency Department, Departmental Pain Therapy Clinic, Azienda Sanitaria Territoriale Ascoli Piceno, San Benedetto del Tronto, ITA

**Keywords:** allodynic pain, chronic pain, neuropathic pain, pain, pens therapy

## Abstract

Introduction: Chronic pain affects millions worldwide, with 15% to 25% being neuropathic due to conditions like diabetic neuropathy and radiculopathy. Neuropathic pain, often described as burning or shooting, includes symptoms like allodynia, significantly impacting the quality of life and posing high economic costs. While pharmacological treatments are common, many patients require more invasive, costly interventions such as spinal cord stimulation. As an alternative, percutaneous electrical nerve stimulation (PENS) therapy (PT) offers a less invasive, cost-effective approach that can be administered outpatient, reducing hospitalization costs and providing psychological benefits like anxiety and stress relief.

Aim: This study aimed to compare the efficacy of PT in the short, medium, and long term in patients affected by chronic neuropathic pain, resistant to conventional pharmacological therapies, using a single active PT probe.

Methods: A total of 100 patients were enrolled for the first PT procedure, and 190 procedures were performed from 2021 to 2023. Excluding those for whom pain could not be detected during follow-up, 41 patients were enrolled, to whom only one PT procedure was performed (Group I). Fourteen patients were enrolled, to whom two PT treatments were performed (Group II), and finally, eight patients were enrolled, to whom three or more PT treatments were performed (Group III). Therefore, a total of 63 patients were enrolled, and 106 PT procedures were performed with pain detected according to the Numeric Pain Scale (NRS) during the entire follow-up period. All patients who presented a trigger point and a specific nevus path or who presented a specific allodynic area caused by a surgical scar were treated.

Results: The group of patients who showed a greater decrease in pain from T0 (baseline) to T1 (three hours after PT) were those who underwent two PT sessions (-52.7%, p = .001). The greatest percentage decrease between before the procedure and seven days after the PT was obtained in the group of patients who underwent three or more PT sessions (T0 4.9 vs. T2 2.9, -40.8%, p < .001). The same group of patients also showed the greatest decrease in pain four weeks after the PT (T0 4.9 vs. T3 3.1, -36.7%, p < .001).

Conclusion: PT appears to be an alternative therapy to pharmacological and infiltrative therapy against chronic neuropathic pain.

## Introduction

Millions of people suffer from chronic pain [1]. Approximately 15% to 25% of chronic pain is neuropathic, with the most common conditions including diabetic neuropathy, post-herpetic neuralgia, and radiculopathy [2]. A systematic review reported an overall prevalence of chronic pain in England of 43.5%, with the rate of moderate to severe disabling pain ranging from 10.4% to 14.3% [3]. Furthermore, the same review reported an increasing trend in chronic pain from 14.3% among 18-25-year-olds to 62% in the over-75 age group [3]. Neuropathic pain, caused by an injury or disease affecting the somatosensory nervous system, also causes allodynia, has a considerable impact on the quality of life of patients, and is associated with a high economic burden for the individual and for society [4,5]. Neuropathic pain can be described as burning, tingling, electric, or shooting and can occur in the absence of a stimulus [6]. Causes of neuropathic pain include nerve injury, infection, metabolic conditions (such as diabetes), and some neurological diseases [7]. There are various pharmacological strategies to reduce pain (e.g., topical therapy, medical therapy, and infiltrative therapy [8,9]. However, very often pharmacological therapies are insufficient to treat it, and invasive interventions are also available to reduce its intensity, such as spinal cord stimulation [10]. In fact, neuromodulation has become an important option for all those traditional therapies that are ineffective [11]. These types of treatment are quite invasive for the patient and very expensive [12,13]. Patients are increasingly turning to non-pharmacological analgesic therapies such as percutaneous electrical nerve stimulation (PENS) therapy (PT). This involves high- and low-frequency electrical stimulation for a period of several minutes via probes inserted through the skin in the area where the pain is perceived [14]. Therefore, peripheral neurostimulation is a cheaper therapy than a surgical approach since it can be performed on an outpatient basis, resulting in a reduction in the costs associated with hospitalization. Furthermore, it can be repeated over time and is less invasive since it uses percutaneous electrodes and does not require the implantation of a permanent device [14]. PT also has several psychological advantages for patients suffering from chronic pain (e.g., reduction of anxiety and stress, and improvement of sleep quality) [15]. However, there are common local adverse effects such as mild discomfort or pain at the application site and/or bleeding, and, in rare cases, infections at the insertion site or nerve damage, which could lead to persistent pain, sensory deficits, or motor dysfunction.

The aim of the study was to compare the effectiveness of PT in the short, medium/long term in patients with chronic neuropathic pain with allodynic/hyperalgesic zones, resistant to conventional pharmacological therapies, using a single active probe.

## Materials and methods

This retrospective study was approved by the Territorial Ethics Center of the Marche (No. 2024-219), and informed consent was obtained from the subjects. A retrospective observational study was conducted at the Departmental Pain Therapy Clinic of the Emergency Department of the Local Health Authority of Ascoli Piceno in Italy. All patients who underwent PT treatment from 2021 to 2023 were enrolled. The inclusion criteria were as follows: patients aged over 18 years, peripheral neuropathic pain with superficial allodynia as defined according to the 2008 International Association for the Study of Pain (IASP) pain terminology [1], chronic pain (>3 months), pain resistant to pharmacological therapy, localized pain, and treatable with a single PENS needle. The exclusion criteria were patients with pacemakers or implantable cardiac defibrillators, local or systemic infections, psychiatric disorders, peripheral body temperature > 37° C, and patients in whom pain could not be detected during follow-up. All procedures were performed on patients admitted to the surgical Day Hospital regime before the outpatient evaluation.

At admission, the following vital parameters were assessed: non-invasive blood pressure, pulse oximetry, ECG, and peripheral body temperature. Patients signed a consent form for treatment. The first step was to identify the peripheral area of the neuropathic pain, followed by locating the trigger point and/or the allodynic and/or hyperalgesic zone. The neuropathic pain was identified on the basis of clinical criteria that differentiated it from other types of pain and diagnosed the lesion or disease potentially causing it. Listening to the patients was a first for the identification of neuropathic pain. Indeed, despite its many causes, neuropathic pain is characterized by the combination of a relatively small number of ‘‘core’’ pain qualities (particularly burning pain, electric shock-like pain, dysesthesia, and brush allodynia) distinguishing it from other types of chronic pain. Subsequently, a physical examination was performed that allowed for the identification of the painful area; when necessary, the examination was conducted using a tool (e.g., Wartenberg neurological pinwheel and/or brush and/or cotton wool) to confirm alterations in the somatosensory system. Immediately before the PT, the area of pain and the zone of greatest pain were identified by asking the patient where they felt the pain and marking the hyperalgesic and/or allodynic area on the body with dermographic markers.

This was followed by the PT treatment with a 21 G probe and neurostimulator. More precisely, the electrode was introduced and tunneled along the pathway of a well-defined nerve target and along the major axis of the painful area, in this last case at a depth between 0.5 and 1.5 cm. After positioning, the probe was connected to the neurostimulator (NeuroStimulator PENS Therapy II®, Algotec Research and Development Limited, Crawley West Sussex, United Kingdom), and the sensory stimulation test program was started (program A at 100 Hz). Once paraesthesia was obtained in the area to be treated, the therapeutic program was set (program C with automatic shifting from 2 Hz to 100 Hz and vice versa every three seconds) at an intensity of 0.5 V for a timing of 25 minutes for all patients enrolled in the study. At the end of the electrical stimulation, the probe was removed, and the patients were kept under observation for three hours and then discharged.

No clinical or technical adverse events were reported. Neuropathic pain was assessed in person using the Numeric Pain Scale (NRS; scale 0-10) (2) before treatment (T0, baseline) and at patient discharge (T1, three hours after PENS). Subsequently, pain was assessed by telephone with the patient at one week (T2) and four weeks after PT (T3). Patients who at T3 had an average pain ≤5 on the NRS and reported a real improvement in the painful clinical condition were then offered a further PT session. Those who had pain ≥5 on the NRS had their pharmacological therapy changed or were offered a new type of pharmacological treatment for the pain.

Statistical analysis

The data were tabulated and analyzed using the IBM SPSS Statistics software version 24 (IBM Corp., Armonk, NY). A preliminary descriptive analysis was carried out using frequencies (n) and percentages (%), central tendency indices (M), and dispersion measures (S.D.). Subsequently, an exploratory analysis was conducted on the differences in the mean levels of the outcomes investigated between the various groups, using the t-test accepting a level of statistical significance p-value ≤ 0.05.

## Results

A total of 100 patients were enrolled for the first PT procedure, and 190 procedures were performed from 2021 to 2023 on these patients. Excluding those in whom pain could not be detected during follow-up, 41 patients were enrolled to whom only one PT procedure was performed (Group I). Fourteen patients were enrolled, to whom two PT treatments were performed (Group II), and finally, eight patients were enrolled, to whom three or more PT treatments were performed (Group III). Therefore, a total of 63 patients were enrolled, and 106 PT procedures were performed with pain detected according to the NRS during the entire follow-up period. Of the patients, 63.40% (n = 26) who received one PT treatment (Group I, Table 1) were female, and the overall sample had an average age of 71.41 years (±14.51). Eight patients were male (57.14%) in Group II (Table 1). Overall, this group had an average age of 62.85 years (±18.40). Finally, six female patients (75%) received three or more PT procedures(Group III, Table 1). The sample overall had an average age of 71.62 years (±15.08) (Table 1).

**Table 1:** Demographic characteristics of the study group Group I: patients who underwent only one PT procedure; Group II: patients who underwent two PT procedures; Group III: patients who underwent three or more PT procedures PT: percutaneous electrical nerve stimulation (PENS) therapy; N: sample size

	Group I	Group II	Group III
Gender (N, %)			
Male	15, 32.60%	8, 57.14%	2, 25%
Female	26, 63.40%	6, 42.86%	6, 75%
Age (Mean, ±SD)	71.41, ±14.51	62.85, ±18.40	71.62, ±15.08

In Group I, 29 patients (70.72%) had a trigger point and a well-defined nerve pathway, and the most affected nerve target was the occipital nerve (n = 15, 36.57%). Still in the same group, those who presented a specific allodynic zone following a surgical scar were 12 (29.28%); of these, three cases had a localization in the lower limbs (7.32%) and three in the right inguinal area (7.32%) (Table 2).

**Table 2:** Nerve pathways and allodynic zones in Group I *Nerve pathways of neuralgia caused by herpes zoster Group I: patients who underwent only one PT procedure; Group II: patients who underwent two PT procedures; Group III: patients who underwent three or more PT procedures PT: percutaneous electrical nerve stimulation (PENS) therapy; N: sample size

Group I					
Nerve pathways of neuralgia			Allodynic zone		
	N	%		N	%
Unpaired ganglion	1	2.44	Lower limb	3	7.32
Occipital	15	36.57	Upper limb	1	2.44
Tibial	6	14.63	Axillary	1	2.44
Trigeminal	1	2.44	Dorsal	2	4.88
Trigeminal V1 and V2	1	2.44	Right inguinal	3	7.32
Trigeminal V2 and V3	1	2.44	Periumbelical	1	2.44
Intercostal*	1	2.44	Vulval-vaginal	1	2.44
Occipital*	1	2.44			
Trigeminal*	1	2.44			
Trigeminal V1*	1	2.44			

Among the patients of Group II, 11 (78.57%) reported a trigger point and a well-defined nervous pathway localized in the trigeminal nerve with its branches (n = 6, 42.86%), which was the most affected nervous pathway. The scar of the lower limb stump (n = 2, 14.29%) was the allodynic zone that was most treated with PT (Table 3).

**Table 3:** Nerve pathways and allodynic zones in Group II *Nerve pathways of neuralgia caused by herpes zoster Group I: patients who underwent only one PT procedure; Group II: patients who underwent two PT procedures; Group III: patients who underwent three or more PT procedures PT: percutaneous electrical nerve stimulation (PENS) therapy; N: sample size

Group II					
Nerve pathways of neuralgia			Allodynic zone		
	N	%		N	%
Occipital	3	21.42	Lumbar	1	7.14
Tibial	2	14.29	Lower limb stump	2	14.29
Trigeminal V1	1	7.14			
Trigeminal V2	1	7.14			
Trigeminal V3	2	14.29			
Trigeminal V1*	2	14.29			

Finally, seven patients of Group III presented a specific trigger point and nervous pathway (e.g., occipital n = 4, 50%), and only one patient (12.5%) was treated for allodynic pain (e.g., back-lumbar surgical scar) (Table 4).

**Table 4:** Nerve pathways and allodynic zones in Group III Group I: patients who underwent only one PT procedure; Group II: patients who underwent two PT procedures; Group III: patients who underwent three or more PT procedures PT: percutaneous electrical nerve stimulation (PENS) therapy; N: sample size

Group III					
Nerve pathways of neuralgia			Allodynic zone		
	N	%		N	%
Occipital	4	50	Dorsolumbar	1	12.5
Trigeminal V1	1	12.5			
Trigeminal V3	2	25			

Mean pain rating according to NRS

As shown in Table 5, the group of patients who had a greater decrease in pain from T0 to T1 were those who underwent two PT procedures (-52.7%, p = .001, Group II). The greatest percentage decrease between before the procedure and seven days after the PT procedure was done in the group of patients who underwent three or more PT procedures (T0 4.9 vs. T2 2.9, -40.8%, p < .001). The same group of patients also showed the greatest decrease in pain four weeks after the PT procedure (T0 4.9 vs. T3 3.1, -36.7%, p < .001).

**Table 5:** Mean pain rating according to NRS Group I: patients who underwent only one PT procedure; Group II: patients who underwent two PT procedures; Group III: patients who underwent three or more PT procedures Group I: T0 vs. T1 and T2 p < .001, T0 vs. T3 p < .05; Group II: T0 vs. T1 p < .001, T0 vs. T2 p < .05, T0 vs. T3 p = .226; Group III: T0 vs. T1, T2, T3 p < .001. Statistical test used: *T-test (p ≤ 0.05 is considered significant) T0: baseline; T1: three hours after PT; T2: one week after PT; T3: four weeks after PT; PT: percutaneous electrical nerve stimulation (PENS) therapy; NRS: Numeric Pain Scale, scale 0 - 10

Group I					Group II				Group III			
	T0	T1	T2	T3	T0	T1	T2	T3	T0	T1	T2	T3
Mean	5.9*	3.2*	4.0*	4.5*	5.5*	2.6*	4.1*	4.6	4.9*	2.4*	2.9*	3.1*
SD	±2.7	±2.6	±2.7	±3.0	±2.9	±2.8	±3.4	±3.4	±3.9	±2.8	±3.0	±3.0

## Discussion

Pain is a subjective and individual experience [16]. It is the result of a complex interaction between the purely sensorial stimulus (painful stimulus) and factors related to the person (e.g. environmental, cultural, religious, emotional, and genetic) that can significantly modify what is perceived [17].

Clinical context and study results

Neuropathic and allodynic pain are often treated with topical pharmacological therapies, such as lidocaine and capsaicin patches. Subsequently, anticonvulsants antidepressants, opioids, and in more severe cases other major opioids are used [18]. In addition, also an infiltrative therapy represented by corticosteroids and nerve blocks with local anesthetics [8,9]. Finnerup et al. show that 40% to 60% of patients with neuropathic pain do not obtain adequate relief with first-line drug therapies [8]. Freynhagen et al. state that infiltrative therapy is used as a temporary treatment for the relief of neuropathic pain and the relief provided by a single infiltration is generally short-lived [19]. In fact, the latter has a limited effect over time with an action that could last up to three/six months [20,21]. Considering the high frequency of chronic pain and the progressive aging of the population, new therapeutic approaches are strongly needed to address this emerging medical need [22]. PT represents a new and valid option for the management of neuropathic pain in all anatomical regions especially for those patients who do not obtain satisfactory results from conventional therapies [23]. Our study shows that the therapeutic effect of PT is effective on pain in a large group of patients with neuropathic pain. Furthermore, to our knowledge, this is the first study in which allodynic zones caused by surgical scars are treated. The prevalence of painful post-surgical scarring is approximately 30% to 50%, while that of burns ranges from 25% to 68% [24,25]. The visible external scar is merely the proverbial tip of the iceberg, as the scar tissue extends beneath the skin and across tissue layers, creating an opportunity for nerve damage or entrapment [26]. Systemic medications (e.g., anti-inflammatories, capsaicin) or topical treatments (e.g., lidocaine patch, capsaicin) can be used as first-line treatments for painful scars, generally before resorting to interventional methods (e.g., PT) [27]. In our study, the three groups of patients treated tended to have a decrease in pain at T1, T2, and T3, as shown in Table 5. Three hours after treatment, all patients in the three groups had almost a 50% reduction in their pain (T0 vs. T1, Group I 45.7%, Group II 52.7%, and Group III 51%, p < .001). Furthermore, the data show that patients who underwent three or more PT (Group III) represent the group where there is the greatest decrease in pain compared to T0 (p < .001). This is also justified by the fact that the T0 evaluation with NRS at each repeated treatment was always lower than the initial evaluation. This last group, as well as the patient group II, needed more treatments despite the positive effect of PT on pain since their painful perception was still a disabling one from a clinical and psychological point of view. In the group of patients who underwent a single PT treatment (Group I), the increase in pain four weeks after treatment was 23.7% (p < .05), among those who received two PT interventions (Group II) the increase was 16.3% (p < .05). Finally, among patients who underwent at least three PT treatments (Group III) the increase was 36.7% (p < .001).

Interpretation of the results and clinical implications

While this reduction in pain is statistically significant, its clinical relevance should be interpreted cautiously. For some patients with chronic and severe neuropathic pain, a 50% reduction may not translate into a meaningful improvement in functional capacity or quality of life. It is well recognized that pain relief alone does not fully address the multifaceted nature of neuropathic pain, which also impacts emotional and psychological well-being. Despite these considerations, the 50% reduction observed in our study represents a statistically significant improvement for the majority of patients, especially in those with severe neuropathic pain who had not responded to conventional therapies. Although these findings are encouraging, they align with previous studies. For instance, Rossi et al. highlighted how PT can significantly reduce pain, with a subsequent increase in pain at follow-up, which is in any case at lower levels than at the initial pre-treatment [23]. Similarly, in our study, this trend was evident, as pain levels increased over time but remained consistently lower than T0 (Table 5). This phenomenon, observed in our study as well, can be attributed to physiological nervous repair, leading to a new increase in the painful stimulus. However, this process remains highly subjective and varies significantly among patients [28].

Therapeutic potential of PT in pain management

The measurement of pain in our study with this time frame would be too limited to be able to compare it to the temporal efficacy of an infiltrative therapy. However, other studies have shown that the average value of pain calculated with the NRS scale at three and six months tends to be equal to that calculated at one month [23]. The highly selective inclusion criteria for the study allowed including groups of patients who presented a specific neurological pain or a defined hyperalgesic/allodynic zone; this highlighted how the effectiveness of the therapy was based on the ability to identify a trigger zone and a specific nerve pathway in the patients to intervene on. This allowed the PENS electrode to be introduced percutaneously in a correct manner, allowing the therapy to be applied directly on the nerve or on the peripheral nerve branches. PT, combined with a low incidence of side effects and the possibility of reducing drug addiction, makes it a valuable treatment in a multimodal approach to the management of neuropathic pain. The clinical benefits are also evident in our sample, in which no complications of any kind were found, either in the short or in the medium/long term (e.g., bleeding and infections). Some studies show that neuropathic pain is associated with a reduction in quality of life and an increase in sleep disorders, depression, and anxiety [29]. Therefore, from the results of our study, we can also deduce a psychological advantage for treated patients that influences carrying out daily activities. In conclusion, our study highlights the potential benefit of PT in the treatment of chronic neuropathic and allodynic pain, particularly in patients who do not respond adequately to conventional therapies. The careful measurement of pain through the NRS scale has proven to be a key factor in guiding access to PT treatment. While these findings are encouraging, further studies are needed to evaluate not only the reduction of painful symptoms but also the long-term effects of PT on patients with neuropathic scar pain. Additionally, future research should focus on the psychological and functional benefits of this treatment, integrating patient-reported outcome measures to provide a more comprehensive understanding of its impact. PT, with its low incidence of side effects and potential to reduce drug dependency, represents a promising component of a personalized, multimodal approach to the management of neuropathic pain. By addressing the complex interplay between physical, emotional, and functional dimensions of pain, PT could play a pivotal role in improving the quality of life for patients with chronic pain.

Limitations

Our study has a few limitations. The exclusive reliance on the NRS for pain assessment evaluates only pain intensity, without accounting for broader impacts on emotional and functional well-being. Moreover, the follow-up period in this study was too short to evaluate the long-term effects of PT, which is particularly relevant in the context of chronic pain management, where sustained efficacy is essential.

## Conclusions

Percutaneous electrical nerve stimulation therapy is an alternative therapy to pharmacological and invasive infiltrative therapy aimed at reducing chronic neuropathic pain. The study shows a positive effect both for the treatment of neuropathic pain in the groups of patients who presented a trigger point and a specific nerve pathway and in the patients who reported allodynia along a surgical scar. Our overall sample showed an average 50% reduction in pain three hours after treatment with a subsequent rise in the curve, indicative of the NRS value at follow-ups, with values in any case lower than the initial pain measurements. Future studies shall need to focus on the effects of PT on pain but also on any benefits it has on the psychology of patients. 
